# Atypical squamous proliferation diagnosed on biopsy has a high risk of representing squamous cell carcinoma

**DOI:** 10.1016/j.jdin.2025.01.008

**Published:** 2025-01-30

**Authors:** Krasimira A. Rozenova, Katelyn A. Reed, Ruifeng Guo

**Affiliations:** a Department of Pathology, Yale School of Medicine, New Haven, Connecticut; b Division of Computational Pathology and AI, Department of Laboratory Medicine and Pathology, Mayo Clinic, Rochester, Minnesota; c Mayo Clinic, Rochester, Minnesota

**Keywords:** atypical squamous proliferation, squamous cell carcinoma, squamous lesion

*To the Editor:* Atypical squamous proliferation (ASP) is a common descriptive pathologic diagnosis, especially in skin biopsies, which does not correlate with a specific clinical entity and encompasses a broad range of squamous lesions with variable outcomes, from benign processes to squamous cell carcinoma (SCC).^1^, ^2^, ^3^, ^4^ The ambiguity of ASP often relates to the challenges of evaluating limited superficial or fragmented biopsy samples, requiring additional tissue sampling for a definitive diagnosis.^1^, ^2^, ^3^, ^4^ Despite the importance of accurate classification of the squamous lesion, it cannot be achieved in many cases. The absence of clear guidance for clinical management post-ASP diagnosis leaves diverse options for follow-up, predominantly at the treating physician’s discretion.^1^, ^2^, ^3^, ^4^ This observational study aims to provide additional data on ASP outcomes based on a retrospective review of 212 patients (218 biopsies) diagnosed with ASP at a large academic center. Our focus is to comprehensively assess ASPs across diverse presentations with an emphasis on clinical outcomes. The cohort included patients with skin and mucosal biopsies diagnosed as ASP. Follow-up data were collected over a median of 55 months (range, 3-201 months). The findings indicate that ASP frequently presents as erythematous plaques or nodules on sun-exposed areas, with the head and neck region being the most common site (47%). Biopsy methods included predominantly shave biopsies (67%), followed by punch (9%) and excisional (5%) techniques (Supplementary Fig 1, available via Mendeley at https://data.mendeley.com/datasets/x4vrpzjb2g/1). Histologically, all biopsies showed irregular squamous acanthosis with varying degrees of cytologic atypia. Of the patients, 20% were immunocompromised, and 59% had a history of nonmelanoma skin cancer, predominantly SCC. Surgical intervention was performed in 43% of cases, including excisions and Mohs surgeries (Table I). Histopathologic re-evaluation revealed that 39% of biopsies with additional tissue samples confirmed a malignant process, aligning with prior studies.^1^, ^2^, ^3^, ^4^ Among the initial classifications, 18% of cases favoring carcinoma, 12% favoring benign, and 12% favoring reactive diagnoses were later confirmed as SCC upon follow-up. Discordance between initial impressions and follow-up diagnoses was observed in 11 cases, emphasizing the importance of re-biopsy for definitive assessment, especially in patients with suspicious clinical features (Fig 1 and Supplementary Fig 2, available via Mendeley at https://data.mendeley.com/datasets/x4vrpzjb2g/1). Immunocompromised patients showed a higher likelihood (16%) of SCC confirmation upon additional sampling, supporting more aggressive management strategies in this subgroup.^5^ In contrast, a prior history of SCC did not significantly influence pathologists’ diagnostic biases. Despite differences in management approaches, the overall risk of ASP recurrence was low, and no cases of metastasis were observed during follow-up.

**Table I:** Summary of demographic data of the patients, biopsy characteristics, and selected relevant clinical history

	Favor reactive	Favor benign neoplasm	Favor malignancy	Neutral	All
Age (median)	70 (range 32-85)	67 (range 34-88)	68 (range 46-97)	67 (range 47-94)	68 (range 32-97)
Male: female ratio	1-1.4	1.5-1	1-1.5	1-1.2	1-1
Biopsies form patients with immunosupression	2 (12%)	19 (28%)	5 (11%)	18 (20%)	44 (20%)
Patients with history of nonmelanoma skin cancer	8 (47%)	46 (71%)	25 (56%)	47 (55%)	126 (59%)
Biopsy site					
Head and neck	4 (24%)	40 (59%)	21 (47%)	38 (43%)	103 (47%)
Upper extremity	3 (18%)	19 (28%)	9 (20%)	23 (26%)	54 (25%)
Lower extremity	8 (46%)	5 (7%)	14 (31%)	21 (24%)	48 (22%)
Trunk	2 (12%)	4 (6%)	1 (2%)	6 (7%)	14 (6%)
Biopsy technique					
Shave biopsy	11 (65%)	57 (84%)	23 (51%)	56 (64%)	147 (67%)
Punch biopsy	2 (12%)	3 (4%)	3 (7%)	12 (14%)	20 (9%)
Excision	1 (5%)	1 (2%)	4 (9%)	4 (4%)	10 (5%)
Unknown	3 (18%)	7 (10%)	15 (33%)	16 (18%)	41 (19%)
Management					
Surgical treatment (including Mohs)	6 (35%)	23 (34%)	25 (56%)	40 (45%)	94 (43%)
Observation only	4 (24%)	13 (19%)	5 (11%)	24 (27%)	46 (21%)
Other	6 (35%)	27 (39%)	10 (22%)	17 (19%)	60 (28%)
No follow-up	1 (6%)	5 (7%)	5 (11%)	7 (8%)	18 (8%)

**Fig 1:**
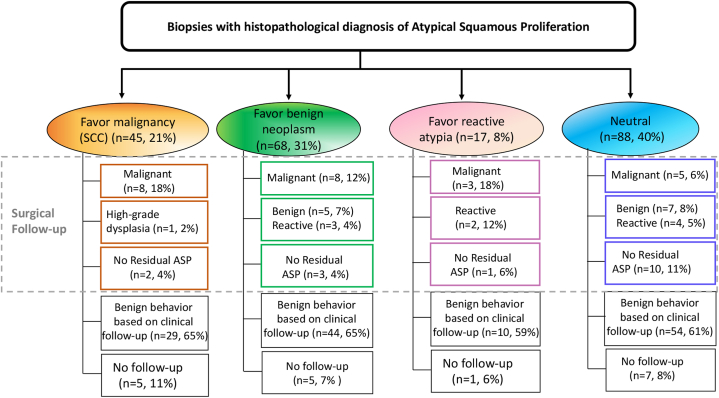
A diagram illustrating 4 categories of ASP biopsies based on the pathologist’s initial impression (ellipses) and the subsequent outcome based on histopathologic findings after surgical re-excision (surgical follow-up) or based on clinical follow-up. A small portion of patients were lost to follow-up. *ASP*, Atypical squamous proliferation.

This study highlights the complex nature of ASP diagnosis and management, emphasizing the role of patient history and clinical context in decision-making. Immunocompromised patients and those with multiple prior SCCs are at increased risk for SCC following an ASP diagnosis, warranting close monitoring. While benign and reactive lesions can mimic ASP, identifying a specific etiology or infectious agent does not preclude the possibility of malignancy. These insights highlight the need for cautious interpretation of ASP in limited biopsies, advocating for repeat biopsies or surgical intervention when initial findings are inconclusive. In conclusion, while many ASP cases resolve benignly, clinicians must remain vigilant, particularly in high-risk populations.

## Conflicts of interest

None disclosed.
